# How Do Older Persons Understand the Purpose and Relevance of Preventive Home Visits? A Study of Experiences after a First Visit

**DOI:** 10.1155/2014/640583

**Published:** 2014-03-10

**Authors:** Mette Tøien, Morten Heggelund, Lisbeth Fagerström

**Affiliations:** ^1^Faculty of Health Sciences, Buskerud and Vestfold University College, Postboks 7053, 3007 Drammen, Norway; ^2^Department of Nursing Science, University of Oslo, P.O. Box 1130, Blindern, 0318 Oslo, Norway; ^3^Hole Volunteer Central, Viksveien 29, 3530 Røyse, Norway

## Abstract

The aim of this study was to explore and describe older persons' experiences of their first Preventive Home Visit. Preventive Home Visits (PHV) are health services that aim to promote older persons' health, prevent functional decline, and reduce the need for comprehensive healthcare. The knowledge base to guide the design of effective PHV interventions is scarce. Studies that explore older persons' experiences of the first visit are essential, as compliance with the service is a prerequisite for positive outcomes. An explorative and descriptive design was applied. Qualitative research interviews with ten older persons who had received the first PHV the previous year were analysed with regard to manifest and latent content. The findings revealed that the understanding of the purpose of PHV varied. For some participants, the concepts and aims of health promotion and disease prevention were difficult to comprehend. The possibility to prepare for the visit was sought. All participants appreciated the service; the dialogue quality was good and a trusted municipal contact person provided security. To enhance compliance and ensure effective PHV, the invitation to the PHV service should include clearly stated aims and specific information about the first visit. An individualised, person-centred approach should be applied.

## 1. Introduction 

By the year 2050, Europe and North America will experience a near doubling of the number of older persons [1]. Taking into consideration both the life quality of the aging population and the expected pressure on healthcare and associated costs, a need exists for healthcare services that aim to promote older persons' health and well-being and prevent or postpone functional decline [2]. Preventive Home Visits (PHV) are one such service. The PHV concept covers a plethora of different, multifaceted interventions, but a shared central component is regular unsolicited visits to independently living older persons aimed to support autonomy and independence, maintain physical functioning, and prevent disability [3]. As opposed to on-demand visits to a General Practitioner (GP), home visits make it possible to reach those not normally seen in the healthcare system [4], to identify and manage risk factors and health problems at an early stage [3, 5], to gain a comprehensive picture of an older person's at-home situation [6], and to meet individual needs that are of importance to an older person's independence [4].

PHV interventions are offered in many developed countries worldwide [7]. Differences between healthcare systems and a lack of evidence based guidelines for how such multifaceted services should be designed and realised have led to substantial heterogeneity within PHV interventions. This includes variation in regard to target population, aim, scope, healthcare visitors' profession and competence and also content, structure, and follow-up strategy [3, 8, 9]. Not all existing services include both health promotive and disease preventive strategies [3, 10]. Randomised Controlled Trials (RCTs) of different PHV interventions have measured various outcomes, and the effects are mixed. Diverse reviews and meta-analyses have indicated a potential for multidimensional PHV interventions to improve several aspects of older persons' health and functional ability [3, 11] and help older persons live safely and independently [8]. Still, due to the heterogeneity of the interventions and in that RCTs are neither adequate nor appropriate for evaluating complex interventions [12]; these reviews and meta-analyses have failed to give clear recommendations for how PHV interventions should be designed and realised to maximise positive outcomes [8, 11, 13].

Qualitative studies are needed to gain a deeper understanding of the active processes and factors that are important to achieving good results [7, 8, 10, 12]. The experiences, attitudes, and perceptions of everyone involved in PHV interventions are essential sources of knowledge, and studies that illuminate older persons' experiences of PHV are especially sought.

Three basic assumptions for effective PHV interventions are related to compliance with the PHV service. The first is that older persons accept the offered PHV service. However, considerable variation exists in regard to initial response [14–16]. It is therefore important to clarify what expectations older persons have of the service and what it will take for PHV to be perceived as being relevant at the start of the intervention [12]. The second is that older persons choose to continue to receive the service after an initial visit, because follow-up over time has shown to improve outcomes [3, 4, 9, 13]. Compliance with PHV services is related to whether the PHV service contributes to meeting individuals' needs [17]. The third is that the effect of PHV lies in older persons utilising the service and actually making changes in their lives that improve health. Qualitative studies show that the relationship between PHV users and the home visitor influences outcomes. This relationship is influenced by the visitor's communication skills, professional competence, caring attitude, ability to implement individualised measures, and ability to understand the coping strategies used by older persons [7, 18]. A synthesis of qualitative home visiting research underscored the importance of building a relationship with and getting to know clients in earlier stages of the visit for the success of specific interventions later on [19]. It is thus important to explore the experiences that older persons have of their first visit regarding relevance, usefulness, and relationship with the visitor; there is reason to believe that these are essential components for further compliance with the intervention. Consequently, the aim of this study was to explore and describe older persons' experiences of their first Preventive Home Visit.

## 2. Materials and Methods 

This study has an exploratory and descriptive design with a qualitative approach.

### 2.1. The PHV Intervention

This study is part of a larger evaluation study of PHV in relation to older persons living in a medium-sized urban Norwegian municipality with about 60000 inhabitants. This municipality has offered PHV for twelve years and has one of the longest experiences with PHV services in Norway. All older persons who live in their own homes and are not recipients of other forms of municipal healthcare services are offered PHV. The main objective of this PHV service is to maintain and promote older persons' health, well-being, and safety so that they can live at home for as long as possible and postpone their need for more comprehensive healthcare services such as home nursing care or nursing home admission [20].

The service is organised under the auspices of municipal home service, and the ten nurses performing the service are responsible for older persons in respective districts. In this municipality, high competence within geriatric nursing and good communication skills is required to work within PHV. Nurses must also be familiar with what legal rights older people have and possess detailed knowledge of relevant services for older persons in the respective districts. In addition to several years of experience within geriatric nursing, all the nurses have relevant formal further education in geriatric nursing, psychiatric nursing or counselling, or a Master's degree in geriatric health care.

When turning 75, inhabitants receive a letter containing brief information on PHV and are offered the service and a calendar date for a first home visit. Those who do not refuse the service are visited, and between 75 and 80% receive a first visit. The aim of this visit is to establish a relationship between visitors and PHV users and to gain a comprehensive understanding of older persons' health and home situations through an open conversation. A thematic guide is applied that includes the following: life history, physical and mental health, functional ability, home safety and need for home modifications, activities, life style, nutrition, family, and social network. Structured survey instruments are not used. During the visit relevant information and support are given; this is intended to empower older persons to utilise their own resources and/or make changes in their daily life. If required, advice to contact their GP or referral to a physiotherapist or occupational therapist is given. Also information and advice on home modification and/or assistive devices are provided. The first visit forms the basis for individualised initiatives and further support. At a minimum, one annual follow-up visit is offered, but further visits may be offered if deemed necessary. The visitor becomes the PHV user's municipal contact person, and users are encouraged to contact their visitor if they have any questions or need help.

### 2.2. Participants

Ten participants were randomly selected from the municipality's list of all PHV users who had received a first visit the previous year and who had not yet received a second visit. PHV users with a diagnosed cognitive impairment were excluded from the list. The list was stratified in order to ensure random selection in the categories of gender and geographical location. A broad and varied sample is considered desirable in qualitative studies [21, 22]. Previous studies have shown differences in the effect of PHV in relation to gender [23]. There is reason to believe that an individual's geographical place of residence can affect his/her experiences of PHV. Different visitors perform the service in different locations, and the municipality is to a great extent divided into socioeconomic neighbourhoods (working class, middle class, or upper class); an individual's socioeconomic status is known to be related to state of health [24, 25]. An administrative employee of the municipality conducted the selection and first contact with the participants. The selection procedure assured the selection of an equal number of men and women and the equal representation of all areas of the municipality. Coincidence rendered the selection equal in regard to whether participants were single or married.

### 2.3. Data Collection

Semistructured interviews were conducted by the second author in the participants' residences during the period from April to June 2011. A thematic guide, formulated with contributions from all the authors, included the following themes: participants' expectations and experiences of PHV, content and structure of dialogues, perceived usefulness of PHV, and suggestions for improvements. The interviewer had rich experience of professional communication with older persons but limited experience with research interviews. To gain interviewing experience and test the interview guide, two taped trial interviews were conducted. After feedback from and discussion with the coauthors, the interviewer's interview skills were approved and small adjustments were made to the interview guide. The semistructured interviews provided the opportunity to obtain the participants' own experiences of PHV. The use of relatively open questions allowed dialogues to focus on what the participants considered important. The thematic guide contributed to the eliciting of answers to the study's aim and ensuring that the dialogues did not become “everyday conversations” [21]. The interviews lasted from 45 to 65 minutes and were digitally recorded and later transcribed verbatim by the second author. To protect confidentiality and anonymity, all data were deleted from the recorder following transferral to the University College's protected research server, and all direct identification information was removed from the transcripts before analysis.

### 2.4. Data Analysis

The data material was analysed with a focus on manifest and latent content within a hermeneutical tradition [26]. Content analysis is the systematic processing and interpretation of a text in order to reveal and describe manifest and latent content [26]. The second and third authors conducted the first steps of the analysis, while all authors contributed to the categorisation and comprehensive interpretation of the data. Even if all direct identification information was removed from the transcripts before analysis, a risk for indirect identification still remained. The decontextualization that occurred during the coding, categorisation, and merging of the categories from all the interviews gradually diminished this risk, and the final report contains no information that can identify participants. To further protect confidentiality during the analysis process, the authors conscientiously stored their work on the research server or on encrypted memory sticks.

Each interview was read through several times by the authors to gain an immediate perception of patterns and themes. The text was thereafter divided into meaning units that were then condensed. The condensed meaning units were subsequently coded, given a short name that described them on a general level. The codes were thereafter sorted into subcategories that formed the basis for categories that described the central meaning content of each interview. Finally, the categories and subcategories from all of the interviews were compared and analysed against one another and then sorted in order to describe the overall manifest meaning content based on the participants' experiences [26], presented in Table 1. Quotations from the interview texts were selected to illustrate and validate the categories. During the final stage of analysis, a comprehensive interpretation of the latent content in the interviews was sought, where the categories and subcategories were viewed in relation to the totality of the interviews and the study's aim [26]. Discussions amongst the authors resulted in consensus on two themes, A and B, also presented in Table 1.

### 2.5. Ethical Considerations

Ethical permission to perform the study was granted from the Regional Committees for Medical and Health Research Ethics (REK) in Norway (number 2011/122b), and the study was conducted in accordance with the Helsinki declaration. Written informed consent from the study participants was given prior to the interviews based on oral and written information about the study. This included information on how confidentiality and anonymity would be safeguarded and the right to withdraw from the study at any time.

## 3. Results 

The results of the content analysis are represented by two overarching themes and related categories and subcategories (see Table 1). Findings related to the participants' understanding of the purpose of PHV are presented first, while findings related to the participants' experiences of PHV are presented second.

### 3.1. Theme A: Older Persons' Understanding of the Purpose of PHV Varies

#### 3.1.1. Older Persons Receive Information and Get a Municipal Contact Person

Many participants experienced that one reason for the visit was the dissemination of information from the municipality. What type of information was given and what they noticed varied according to the older persons' different needs. One participant stated: “*It was a straightforward briefing we received information on how we should take care of ourselves by eating regularly and well*.”

It was important for several participants to be given a municipal contact person to turn to if needed. For some it was important to have a mediator who could engage other municipal services; for others it was practical help in procuring assistive devices when needed. The most important thing for the majority of the informants, nevertheless, was the sense of security that came from having a contact person. One participant said: “*It is great for us to have a contact to call rather than to call a switchboard should there be something. It is reassuring*.”

#### 3.1.2. Assessment of the Older Person's Health and Environment

The assessment of health and health status was perceived as a significant area of focus during the visit. Some participants related that they spoke about their health situation while others said that they were engaged in telling their life history. As one participant stated: “*We talked a lot about me and what I have gone through in life.*” Yet another participant said: “*She asked if we had any problems and wanted to know our status.*” Still others experienced that the conversation revolved around everything from an early assessment of dementia to a conversation to reveal difficulties in their lives.

Also the assessment of the living environment was a central theme. Some participants said that the conversation focused on altering the house and assistive devices or how one could stay in one's own home for as long as possible, while others mentioned that the conversation was about changing residence. One participant said: “*I think it is good that persons are encouraged to manage at home.*” The conversations could also be about safety in the home: “*She talked about loose cords, rugs and what (kind of) bathroom we have, and whether we had a bathtub.*”

#### 3.1.3. Overview of Older Persons' Health and Home Situation for Health Care Leaders

Several participants recounted that the visit was intended to identify and assess their various needs. Some believed that the home visit nurse would record their situation and then pass this information along to the authorities in the municipality. One described it thus: “*The purpose of the visit was that the municipality wants to map the elderly so that they have a better overview of them.*”

Some participants experienced the visit as being mandatory. As one said: “*It was probably from the municipality's side to get an overview of what you can expect in the future, what the condition is, and how it is for me*.” Some participants described the visit as an inspection or a compulsory visit from health services. As one participant stated: “*She probably wanted to have the conversation in order to keep her system organised and that was fine*.”

#### 3.1.4. Help and Support for Health and Independent Living

Several participants recollected good and informative conversations about health related themes. One said: “*There was talk about injections for the flu and infection control, we got to know where the infection control office was.*” Others mentioned that they are now more aware of everyday risk factors after the visit. As a participant stated: “*I have become more aware of this with slippery stairs, think more about eating properly with more fruit and vitamins.*” Several participants experienced that they received help with various initiatives during the visit, such as assistive devices, exercise groups, social groups, or travel for older persons. Some participants experienced that they did not need any help during the visit but that they valued knowing that help was available for the day they might need it.

#### 3.1.5. Unclear Perception of Offer

Several participants expressed that the service was related to help with a preexisting condition, disease, or functional disability, and they perceived the service to be unnecessary in that they felt that their everyday life ran smoothly. One participant commented: “*I do not have a need for the visit, feel well and fit*”. Another commented: “*We had no express need we could discuss*”. Yet another participant summed up by saying: “*The sum of the entire conversation is that at present I do not have a use for any of the services but it is good to know that it is there*”.

The participants also had differing expectations of what the visit would focus on. One participant commented: “*I am a little mean when I say that, but she showed (me) a brochure of older persons who were walking with a walker and then I shuddered a bit.*” Other participants described that they hoped for short visits in that there was nothing special to discuss, while others did not want more than one visit. One participant said: “*I called and told them that we were doing well and that she could drop the second visit for the benefit of those who perhaps had more use for it instead of us*.” Another participant stated: “*I do not want someone who (comes here) once a year to see if there has been a change from last year in the house or body*.”

Some participants expressed a desire to prepare for the visit. As one stated:

*What could have made such a home visit even better was if I had received some of the questions ahead of time so that I would have been clear about what we would discuss, so it was not so much (just) a nice chat. *

Another participant said: “*She will be back in a year, it is fine then we will to be sure be more prepared in that way that we can know what we should talk about.*”

### 3.2. Theme B: A Good and Trusting Relationship

#### 3.2.1. A Good Dialogue

Several participants had a positive experience of the visit. They were happy about it and considered it a positive initiative. Several experienced that they had a good conversation with the home visit nurse who conducted the visit. One commented: “*I was very anxious, but it was a nice lady and we conversed well together.*” Some participants related that the visit lasted several hours and that the conversation flowed freely without seeming to last too long. The vast majority expressed a sense of security about being followed up on in case their daily life should become difficult. Participants also appreciated the opportunity to get in touch if needed, as one participant expressed: “*I can trust that if I called and needed help then I would receive a visit.*”

Many participants said their willingness to speak freely about their health, life, and everyday life depends on a sense of trust in the visitor: “*It must be a person you have confidence in and have a certain chemistry with.*” One participant concluded that she talks about herself provided that she feels she can make a connection with the visitor and that the visitor responds to what she is saying. Almost none felt that it was difficult to talk about him/herself with the visitor.

#### 3.2.2. Varying Needs

Even though the majority of the participants were relatively fit and well-functioning, their health status and living conditions varied. This was reflected in their expressed needs and experiences of help from the PHV visitors described in the previous sections.

For some participants, PHV starting at age 75 was considered too early, while for others it was deemed to be timely. Several participants considered that the visits should be realised earlier for those who were in a frail state or who had a limited social network. “*Some perhaps need a visit earlier who are (in poorer health) and have difficulties getting out or who do not have a family to turn to.*”

### 3.3. Summary of Findings

The participants reported several different perceptions of the purpose of PHV, and the purpose therefore was somewhat unclear. Participants' perceptions were influenced by what they experienced as being challenging in everyday life. Some reported that they had no need for the PHV intervention in that they were healthy and managed well.

All of the participants, however, had a positive experience of their first visit. The participants experienced that they developed a good and satisfactory relationship with a municipal representative and that the service simultaneously contributed to their sense of security. Several participants reported a good experience of the conversation that took place during the visit. They had confidence in the home visit nurse and experienced that she was easy to speak to, which enabled them to build a relationship with her. Nonetheless, some participants did not identify with all of the themes presented during the visit. Several sought visits that were tailored to individual needs and the possibility to prepare for the visit.

## 4. Discussion

The stated purpose of the municipality's PHV intervention was to contribute to the promotion of older persons' health, well-being, and security and support their ability to live at home for as long as possible [20]. The participants in this study had various perceptions of the purpose of PHV. This may indicate that the information sent out prior to the visit and/or the focus during the visit was perceived as somewhat ambiguous. It could also be that not every participant had read the information. Despite a somewhat unclear understanding of the purpose of the PHV service, only a relatively small number refused the service in this municipality. One explanation for such can be that the municipality followed research-based recommendations for the invitation procedure [27]. Another explanation for the high acceptance rate may be that the service was well established and had a good reputation amongst older persons in the municipality.

A number of participants said that they did not have such a great need for the service in that they were healthy and functioning well in everyday life, while other participants stated that some of the dialogue themes were not relevant to the daily life they lived. It might be that some participants misunderstood and thought that the PHV service was ordinary home nursing care, which is based on the need for care or treatment of actual health problems and disease. The fact that both services are provided by nurses may have increased confusion. This may also indicate that some participants had not fully understood the intention behind PHV, which is to help maintain good health well before an individual becomes frail. If older persons do not understand what health promotive and disease preventive initiatives can contribute and do not identify themselves with potential risk factors, it becomes difficult to reach them with recommendations for home modifications or lifestyle changes [28, 29]. Being too healthy is reported as a reason for rejecting PHV service in two quantitative PHV studies [30, 31]. Our findings are therefore important because understanding the intention behind PHV is central both to acceptance of the service and for further compliance with PHV. Regardless, it is important that older persons can and are given the opportunity to influence the content of PHV and that home visit nurses bolster and support older persons in discovering their own resources and limitations so that they can be equipped to face difficulties in old age [32, 33].

The finding that some participants perceived that compliance with the PHV service was mandatory and that the purpose was to assess the health status and at-home situation of older persons living in the municipality was similarly worrying. It is both legitimate and important for the municipality's authorities to seek such an overview in order to be able to plan future healthcare. However, if PHV are to yield a positive result regarding the health of older persons and their ability to live at home, the focus must lie on supporting each person's individual needs and resources [8, 17], and follow-up over time has been shown to increase the probability of effectiveness [4, 9, 13]. If PHV users perceive that the delivery of information is the aim of the visit, this focus could inhibit their asking questions related to own health and future situation. Such a view could therefore constitute an obstacle to the utilisation of the visitors' competence in regard to health promotive and disease preventive initiatives and could reduce further compliance with the PHV service. A lack of clarity regarding the purpose of PHV can, accordingly, result in the inability of older persons to receive the full benefit of the intervention.

Some participants expressed the desire to prepare for the visit. This is an important new finding because this indicates that the possibility exists to adjust the approach to these services and thus enhance PHV users' engagement with, and utilisation of the service. Detailed and clear information will help contribute to the clarification of what the visit will be about and allow participants the possibility to reflect on their situation at leisure and prepare for the visit, helping ensure it is perceived as being relevant and important for each individual.

Despite unclear expectations of what the visit would entail, the participants were satisfied and had a good experience of the visit. Several participants expressed that the purpose of the visit was to help solve everyday challenges regarding health and independent living. The alteration of a residence or assistive devices can compensate for failing functional ability and make it both easier and possible to maintain independent living [34]. Additionally, help in relation to social challenges was reported, such as a referral to various groups and/or the establishment or broadening of a participant's social network. Loneliness is common amongst older persons and correlates with adverse mental and physical health consequences [35, 36]. Personalised initiatives aimed at social contacts can, thus, result in significant health benefits.

Several participants mentioned that the visit was meant to ensure that older persons were given a municipal contact person. Many emphasised that they felt a sense of security having been given a person to contact if something unexpected should occur. Especially for those with a limited social network, this could comprise a reassuring factor in their everyday life. This is consistent with the results seen in a Swedish study [37]. Antonovsky maintains that an individual's conditions for mastery of his/her situation and maintaining health are dependent on the individual experiencing life as comprehensible and predictable and having confidence in that resources are available for mastering meaningful challenges [38]. Consequently, for older persons living at home this sense of security and experience of being supported can, when life becomes difficult, improve mastery and positively influence health.

Participants appreciated the good dialogue with the visitor. A high level of competence in relation to communication and counselling is significant in regard to PHV [18]. PHV have an advantage over other preventative initiatives in that they take place in the older person's home, something that naturally creates a safe framework for dialogue and makes the individual more at ease and willing to talk [37]. Many participants emphasised the importance of having a good and trusting relationship with the visiting nurse so that they could talk freely about their lives. The importance of a home visit nurse's personal resources in regard to how an older person experiences the visit is also seen in other studies [7, 18]. Fagerström et al. found that a good relationship with the home visitor is necessary to initiate a dialogue on needs and health resources [10], and it has also been found that the initial establishment of a trusting relationship is essential to realising initiatives later on [19]. The perceived sense of trust in the visitor found in this study therefore constitutes a precondition for PHV as a health promotive and disease preventive service.

### 4.1. Methodological Considerations

The selection procedure was planned to secure a so rich and varied data material as possible, yet the preponderance of resource-strong older persons agreeing to participate in the study may limit the transferability of the findings. Another limitation could be that the interviewer had limited experience of conducting semistructured interviews, which may have affected the quality of the interviews, especially in regard to the ability to elicit depth and breadth in the participants' reflections on the different themes. Two authors performed the initial analysis and all the three authors participated in the final stages of the analysis, which enhanced rigour and trustworthiness.

## 5. Conclusion 

This study demonstrates the necessity of exploring older persons' experiences and perceptions of PHV in order to be able to develop the service so that it truly responds to individual needs and preferences. The study shows that the concepts and aims of health promotive and disease preventive interventions can be difficult for older persons to comprehend, which may influence compliance and the effect of PHV. Information about the purpose and goal of PHV—both in terms of preventing disease and what it entails to promote health and make living at home possible—must therefore be clearly formulated in the advance information given, and each individual older person's understanding of the purpose of PHV should be investigated and, if needed, corrected during the first visit. The advance information should also contain specific information that enables PHV users to prepare for the visit.

PHV performed by experienced and well-educated nurses can contribute to increased security for older persons living at home and an increased awareness of the ability to influence one's own health and housing situation at an advanced age. Dedicated and competent nurses have the ability to lay the groundwork for a trusting relationship, as a basis for further constructive cooperation. In order for PHV to be perceived as being relevant, it is important to adapt the content of the PHV dialogue to older persons' needs and preferences. A person-centred perspective is therefore essential to increasing the efficiency of PHV and ensuring effective and good results.

## Figures and Tables

**Table 1:** Older persons' experiences of their first Preventive Home Visit.

Theme	Category	Subcategory
A: Older persons' understanding of the purpose of PHV varies	Older persons receive information and get a municipal contact person	Older persons receive information
		Older persons get a municipal contact person
	Assessment of the older person's health and environment	Assessment of health
		Assessment of residence
	Overview of older persons' health and home situation for health care leaders	Registration of health and home situation
		Registration of need for services
	Help and support for health and independent living	Health promotive and disease preventive initiatives
		Offer of help
	Unclear perception of offer	Unclear perception of purpose
		Little possibility to prepare

B: A good and trusting relationship	A good dialogue	Contact person provides security
		Trust in the visitor
		Positive experience of offer
	Varying needs	Varying health status and living conditions
		Timing of the visit

## References

[1] World Health Organisation and US National Institute of Aging (2011). *Global Health and Aging, World Health Organisation and US National Institute of Aging*.

[2] Christensen K, Doblhammer G, Rau R, Vaupel JW (2009). Ageing populations: the challenges ahead. *The Lancet*.

[3] Markle-Reid M, Browne G, Weir R, Gafni A, Roberts J, Henderson SR (2006). The effectiveness and efficiency of home-based nursing health promotion for older people: a review of the literature. *Medical Care Research and Review*.

[4] Vass M, Avlund K, Hendriksen C, Philipson L, Riis P (2007). Preventive home visits to older people in Denmark: why, how, by whom, and when?. *Zeitschrift für Gerontologie und Geriatrie*.

[5] Hendriksen C, Lund E, Strømgård E (1984). Consequences of assessment and intervention among elderly people: a three year randomised controlled trial. *British Medical Journal*.

[6] Vetter NJ, Jones DA, Victor CR (1986). A health visitor affects the problems others do not reach. *The Lancet*.

[7] Sahlén K-G (2009). *An Ounce of Prevention Is Worth a Pound of Cure: Preventive Home vIsits Among Healthy Seniors*.

[8] Beswick AD, Rees K, Dieppe P (2008). Complex interventions to improve physical function and maintain independent living in elderly people: a systematic review and meta-analysis. *The Lancet*.

[9] Stuck A, Kane RL (2008). Whom do preventive home visits help?. *Journal of the American Geriatrics Society*.

[10] Fagerström L, Wikblad A, Nilsson J (2009). An integrative research review of preventive home visits among older people—is an individual health resource perspective a vision or a reality?. *Scandinavian Journal of Caring Sciences*.

[11] Gustafsson S, Edberg A-K, Johansson B, Dahlin-Ivanoff S (2009). Multi-component health promotion and disease prevention for community-dwelling frail elderly persons: a systematic review. *European Journal of Ageing*.

[12] Clark J (2001). Preventive home visits to elderly people. *British Medical Journal*.

[13] Frost H, Haw S, Frank J (2012). Interventions in community settings that prevent or delay disablement in later life: an overview of the evidence. *Quality in Ageing & Older Adults*.

[14] Sahlen K-G, Dahlgren L, Hellner BM, Stenlund H, Lindholm L (2006). Preventive home visits postpone mortality—a controlled trial with time-limited results. *BMC Public Health*.

[15] Vass M, Avlund K, Hendriksen C, Andersen CK, Keiding N (2002). Preventive home visits to older people in Denmark: methodology of a randomized controlled study. *Aging-Clinical & Experimental Research*.

[16] Wyller TB, Pettersen AM (2005). *Forebyggende hjemmebesøk til eldre: Norge—med blikk mot Sverige og Danmark: en kartleggingsundersøkelse [Preventive Home visits to older persons in Norway -Looking towards Sweden and Denmark]*.

[17] van Haastregt JCM, Diederiks JPM, van Rossum E, De Witte LP, Crebolder HFJM (2000). Effects of preventive home visits to elderly people living in the community: systematic review. *British Medical Journal*.

[18] Yamada Y, Vass M, Hvas L, Igarashi A, Hendriksen C, Avlund K (2011). Collaborative relationship in preventive home visits to older people. *International Journal of Older People Nursing*.

[19] McNaughton DB (2000). A synthesis of qualitative home visiting research. *Public Health Nursing*.

[20] Municipality of Drammen (2009). *Forebyggende helseteam for eldre [Preventive health team for older people]*.

[21] Kvale S, Brinkmann S (2008). *InterViews: Learning the Craft of Qualitative Research Interviewing*.

[22] Polit DF, Beck CT (2012). *Nursing Research: Generating and Assessing Evidence for Nursing Practice*.

[23] Vass M, Avlund K, Kvist K, Hendriksen C, Andersen CK, Keiding N (2004). Structured home visits to older people. Are they only of benefit for women? A randomised controlled trial. *Scandinavian Journal of Primary Health Care*.

[24] Eikemo TA, Huisman M, Bambra C, Kunst AE (2008). Health inequalities according to educational level in different welfare regimes: a comparison of 23 European countries. *Sociology of Health and Illness*.

[25] Marmot M (2005). Social determinants of health inequalities. *The Lancet*.

[26] Graneheim UH, Lundman B (2004). Qualitative content analysis in nursing research: concepts, procedures and measures to achieve trustworthiness. *Nurse Education Today*.

[27] Ekmann A, Vass M, Avlund K (2010). Preventive home visits to older home-dwelling people in Denmark: are invitational procedures of importance?. *Health and Social Care in the Community*.

[28] Carter KF, Kulbok PA (2002). Motivation for health behaviours: a systematic review of the nursing literature. *Journal of Advanced Nursing*.

[29] Conn VS, Hafdahl AR, Mehr DR (2011). Interventions to increase physical activity among healthy adults: meta-analysis of outcomes. *American journal of public health*.

[30] Minder CE, Müller T, Gillmann G, Beck JC, Stuck AE (2002). Subgroups of refusers in a disability prevention trial in older adults: baseline and follow-up analysis. *American Journal of Public Health*.

[31] Vass M, Avlund K, Hendriksen C (2007). Randomized intervention trial on preventive home visits to older people: baseline and follow-up characteristics of participants and non-participants. *Scandinavian Journal of Public Health*.

[32] Hage AM, Lorensen M (2005). A philosophical analysis of the concept empowerment; the fundament of an education-programme to the frail elderly. *Nursing Philosophy*.

[33] Vass M, Avlund K, Lauridsen J, Hendriksen C (2005). Feasible model for prevention of functional decline in older people: municipality-randomized, controlled trial. *Journal of the American Geriatrics Society*.

[34] Wahl H-W, Fänge A, Oswald F, Gitlin LN, Iwarsson S (2009). The home environment and disability-related outcomes in aging individuals: what is the empirical evidence?. *Gerontologist*.

[35] Luanaigh CÓ, Lawlor BA (2008). Loneliness and the health of older people. *International Journal of Geriatric Psychiatry*.

[36] Barg FK, Huss-Ashmore R, Wittink MN, Murray GF, Bogner HR, Gallo JJ (2006). A mixed-methods approach to understanding loneliness and depression in older adults. *The Journals of Gerontology: Series B*.

[37] Sherman H, Karp A, Blid SS, Wånell  SE (2010). Health dialogues with 75-year olds in their homes—an important part of the community nurse’s preventive work. *Socialmedicinsk Tidskrift*.

[38] Antonovsky A (1996). The salutogenic model as a theory to guide health promotion 1. *Health Promotion International*.

